# Accurate Visuomotor Control below the Perceptual Threshold of Size Discrimination

**DOI:** 10.1371/journal.pone.0036253

**Published:** 2012-04-27

**Authors:** Tzvi Ganel, Erez Freud, Eran Chajut, Daniel Algom

**Affiliations:** 1 Department of Psychology and Zlotowski Center for Neuroscience, Ben-Gurion University of the Negev, Beer-Sheva, Israel; 2 Department of Education and Psychology, The Open University of Israel, Raanana, Israel; 3 Department of Psychology, Tel-Aviv University, Tel-Aviv, Israel; The University of Western Ontario, Canada

## Abstract

**Background:**

Human resolution for object size is typically determined by psychophysical methods that are based on conscious perception. In contrast, grasping of the same objects might be less conscious. It is suggested that grasping is mediated by mechanisms other than those mediating conscious perception. In this study, we compared the visual resolution for object size of the visuomotor and the perceptual system.

**Methodology/Principal Findings:**

In Experiment 1, participants discriminated the size of pairs of objects once through perceptual judgments and once by grasping movements toward the objects. Notably, the actual size differences were set below the Just Noticeable Difference (JND). We found that grasping trajectories reflected the actual size differences between the objects regardless of the JND. This pattern was observed even in trials in which the perceptual judgments were erroneous. The results of an additional control experiment showed that these findings were not confounded by task demands. Participants were not aware, therefore, that their size discrimination via grasp was veridical.

**Conclusions/Significance:**

We conclude that human resolution is not fully tapped by perceptually determined thresholds. Grasping likely exhibits greater resolving power than people usually realize.

## Introduction

A fundamental (hence rarely articulated) feature of psychophysics is that it usually involves the conscious evaluation of the stimuli impinging upon the senses [1], [2]. After all, people can only judge stimuli and attributes that they are consciously aware of. Here, we tested the processing of object size, applying psychophysical measures to compare conscious perception of objects to visuomotor interactions when grasping these objects. It has been argued that the processes mediating conscious perception and visuomotor control are based on distinct neural mechanisms and that people might be less conscious when they reach with their hands for an object than when they merely report its perceived size [3], [4].

The most intriguing – yet also the most controversial evidence – for dissociations between action and perception in healthy subjects has come from studies of visual illusions (for reviews see [5], [6]). Visual illusions, by definition, have robust effects on perceptual judgments but have been argued to have little or no effect on visuomotor control. Thus, unlike perceptual tasks, the opening of the grasping hand is, in most cases, unaffected by the visual illusions when people reach out to pick up objects embedded in illusory displays. This evidence suggests that the hand is not deceived by the same information that deceives the eye [4]. However, this interpretation has been vigorously challenged over the past decade by studies reporting inconstant findings of illusory effects on grasping [7].

In a recent study [3], we presented evidence for double dissociations between perception and grasping with respect to a well-known visual illusion, the Ponzo illusion. In this illusion, a pair of equally long lines is perceived to be of unequal length due to (irrelevant) contextual cues associated with linear perspective. In the context of the Ponzo illusion, the results showed that even in trials in which the participants erroneously perceived the larger stimulus in the pair to be the smaller one, their fingers trajectories during grasping were tuned to reflect the true physical size differences between the objects. The trajectories of the fingers during grasp can therefore be dissociated from people's conscious perception of size [3], [8].

More recent findings from our laboratory showed that Weber's law, a pillar of classical psychophysics, does not necessarily hold when people grasp rather than perceptually evaluate the size of objects presented for view [9], [10]. We derived the JND for grasping and for perceptual estimation for a range of object sizes. For perceptual estimations, the JND linearly increased with object size, supporting Weber's law. Startlingly, the JND remained constant during grasping (at the time of peak grip aperture), violating Weber's law.

The current study sets out to provide a framework to study potential differences between the way visual size information is processed by the visuomotor and the perceptual systems. To this end, we tested for possible differences in visual resolution to size between the visuomotor system and the perceptual system. Although research aimed at studying the resolution of the perceptual system is well rooted in the field of psychophysical science [11], little research has focused on the resolution of the visuomotor system [12]. To best of our knowledge, the resolution of the two visual systems has not been directly compared within the same paradigm. The purpose of the current study was to test whether visual resolution within a given size range can be dissociated for grasping and perception.

In Experiment 1, the participants were presented with a pair of different-size stimuli on each trial. They were asked (a) to grasp one of the stimuli and (b) to compare the pair perceptually on size. Because the stimuli were fairly close in size and were set below JND, an appreciable portion of the perceptual judgments were erroneous. The focus of interest was the sensitivity of the grasping fingers on the incorrectly discriminated trials. Does differential sensitivity to grasping exist when differential sensitivity to vision is absent?

We set the physical size difference between the pairs of stimuli to be just below the JND. The value of the physical difference separating the pair of stimuli was determined based on the relevant psychophysical studies [1], [13], [14]. Our tactic in this study was to expose a single pair of stimuli was for extensive measurement. The current size difference of about 1% was well below the agreed estimation of the Weber fraction for visual length (3%, see [15]). The location of the two stimuli altered across trials and the participant made a perceptual judgment (“larger or smaller”) with respect to size in each trial. In order to reduce the demand characteristics, we asked the participant to grasp only one of the stimulus objects on each trial (for a similar design, see [3]). This did not pose a problem for data analysis (comparing the Maximum Grip Aperture, MGA, during grasping for the two stimuli and relating the MGA to the psychophysical comparison).

Again, apart from overt judgments of relative size, the participants were also asked to grasp one of the stimuli while their fingers' position was tracked. This allowed us to test the resolution power of the grasping fingers with stimuli of barely detectable difference in size. Does grasping resolution depend on people's conscious judgment of object size?

To anticipate the results of Experiment 1, we found that grasping resolution of size did not interact with perceptual resolution of size. The purpose of Experiment 2 was to test if this surprising dissociation has resulted from differences in the nature and measurement of the pertinent tasks. In particular, grasps have been analyzed using a continuous, sub-mm accuracy measure, whereas perceptual judgments were limited to a dichotomous 2-choice size discrimination. Therefore, in Experiment 2, we replaced grasping by a continuous measure of perceived size, accomplished by reaching out and positioning the finger and the thumb at a distance that is perceived to be that of the size of the target object. Notably, the participant did not grasp the object itself. Notably, too, despite the engagement of the fingers, this manual size estimation is based on perception [3], [8], [9]. These perceptual-manual estimations provide a continuous measure of perceptual resolution with the same effectors used for grasping (Experiment 1).

Therefore, Experiment 2 entailed two perceptual responses to size. One was that of the dichotomous size discrimination from Experiment 1 and the other was the continuous estimation of size. Because both measures are perceptual, we expected to find an interaction between the two, unlike the case in Experiment 1. In particular, we predicted accurate manual estimations for correct size classifications but not for erroneous classifications.

## Materials and Methods

### Experiment 1

#### Participants

Twenty eight right-handed undergraduate students received course credit for their participation in the experiment. Handedness was assessed by a modified version of the Edinburgh Handedness Inventory [16]. The experimental protocol was approved by the local ethics committee.

#### Apparatus and stimuli

**Figure 1:**
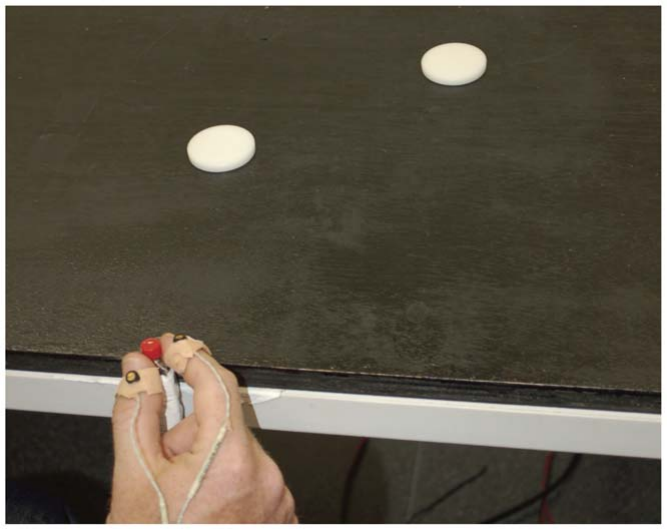
An illustration of the experimental setup. Subjects were asked to make explicit size comparisons between two disks (40 mm and 40.5 mm in diameter). Within the same trials, subjects were also asked to grasp the central target disk. The 3D position of the fingers was tracked by three infra-red light emitting diodes attached to each participant's index finger, thumb, and wrist. This design allowed us to compare grasping resolution between correct and incorrect perceptual judgments trials.

Participants were sitting in front of a black tabletop on which two objects were placed at a viewing distance of approximately 40 cm (see Figure 1). Computer-controlled PLATO goggles (Translucent Technologies, Toronto, ON) with liquid-crystal shutter lenses were used to control stimulus exposure time. Grip scaling was recorded by an Optotrak Certus device (Northern Digital, Waterloo, ON). The apparatus tracked the 3D position of three active infra-red light emitting diodes attached separately to the participant's index finger, thumb and wrist. This allowed for complete freedom of movement of the hand and fingers. Note that the precision grasps were always performed using the tip of the finger and the thumb, whereas the actual placing of the diodes was on the nail side of the finger and the thumb. In particular, the diodes were placed at the midpoint of the inner nail-finger border of thumb and forefinger (see Figure 1). The arrangement meant to avoid interference with natural grasping. This placement of the diodes, conventionally used in grasping studies, results in an artificially inflated value of the grip aperture due to the inclusion of finger's width, which was corrected for in our post analysis of the data (see Data Analysis section). Prior to each grasp, subjects were asked to hold a cylindrical (18 mm in diameter) “start” button. This was done to ensure identical starting conditions for all grasps. This explains why the measured difference between the fingers prior to grasping reflected the size of the start button.

A 200 Hz sampling rate was used for the Optotrak, which provides a 0.1 mm positional accuracy under the experimental conditions used. The target objects were two 1 mm thick circular disks. The smaller disk was 40 mm in diameter and the larger disk was 40.5 mm in diameter. This difference in size between the two disks was estimated to be below the reported values of JND in the literature. The goal was to generate a sizeable number of erroneous (and correct) perceptual judgments to be compared with the pertinent outcome of grasping. To minimize the possibility that participants identify the disks using irrelevant superficial features, two different exemplars were used for each of the disks.

#### Experimental Procedure

One of the objects (the target object for grasping) was placed about 15 cm in front of the participant's initial hand position. The second object was placed 10 cm to the right or the left (lower or upper corner) side of the target object (see Figure 1). Object sizes (target object smaller or larger) and objects positions were counterbalanced across trials. In each trial, the participant was asked (a) to grasp the object in central position and (b) to report which of the two objects was larger in size (for a similar design, see [3]). The within-trial order of perceptual discrimination and grasping was counterbalanced so that, within each trial, half of the participants completed the perceptual task before the grasping task whereas the other half performed in the converse order. Perceptual estimations in this condition were performed after participants have completed each grasp and moved back to the start position. Following a short practice and equipment-calibration, each subject performed 3 consecutive experimental blocks (32 stimulus presentations in each block with short breaks between the block) during the grasping experiment. For perceptual discrimination, the participant indicated whether the central object was larger or smaller than the peripherally placed one. For grasping, the participant was asked to grasp the central object and to place it back then to its initial position. On each trial, the goggles opened for 2 s (followed by a short auditory beep) during which the participant made either a verbal judgment of relative size or started to grasp the central object. Following a 500 ms interval, a second auditory beep indicated the time to perform the second response (perceptual judgment or grasping) for which additional 2 s were allowed. The goggles were then closed prior to the initiation of the following trial.

#### Data analysis

On each trial, we recorded the 3D trajectories of the fingers during grasp. Movement onset was defined as the point where the velocity of the finger was above 25 mm/s for 10 consecutive frames (50 ms). Movement offset was defined as the first frame where changes in grip aperture were smaller than 0.2 mm for a continuous period of 25 ms. We have then computed fingers aperture and between-target difference scores (large target minus small target) at 10% increments in movement time. To correct for fingers width, we used the following method that has the advantage of being independent from the initial aperture between the fingers. First, final grip aperture (100%, which includes the fingers width as well as the physical object size) was computed for each trial. We then subtracted, for each trial, a constant value reflecting the physical size of the smaller object (40 mm) from the block average 100% aperture (Note, that if we would have subtracted the corresponding object size, 40 or 40.5 mm, from each trial, this would have resulted in artificial elimination of the effect of object size). The result gave us a reliable measure of the finger's width. We have then subtracted the corresponding fingers width from each data point (0–100%) for each trial.

Of more importance, we determined in each trial the maximum grip aperture (MGA) between the finger and thumb. The MGA, together with the anticipatory course of the fingers prior to grasp, are known to be well correlated with the size of the target object [12]. The MGA was the main dependent variable of interest in this study. To correct for fingers width, we used the measure described above and subtracted from each data point of each of the subjects their average finger width. Six subjects were removed from the analysis due to missing data in one or more of the experimental conditions.

### Experiment 2

#### Participants

Thirty right-handed undergraduate students received course credit for their participation in the experiment.

#### Experimental Procedure

The experimental procedure was similar to that of Experiment 1 with the notable exception that the grasping task was replaced by a manual estimation task. The participants were now asked to make psychophysical estimates of length by opening their index finger and thumb to match the size of the stimulus in a position located about five centimeters to the right of the starting position. Therefore, the same kinematic information (tracked using the Optotrak design) was available for both the grasping and the estimations experiments. Participants were also asked to grasp each object immediately *after* completing their estimation responses. This was done to ensure that they received the same tactile feedback in the estimation and in the grasping experiments. As in Experiment 1, participants were also asked to make a two-choice size discriminations of the two stimuli prior (or after) their size estimations. The within-trial order of perceptual discrimination and manual estimation was counterbalanced as in Experiment 1.

#### Data analysis

The data analysis was similar to that in Experiment 1, with the exception that the main data-set of interest was the point in time in which participants made their manual estimation response (when estimating size, participants were asked to steadily hold their estimation position for approximately one second). Estimation aperture was set as the first frame where changes in grip aperture were smaller than 0.2 mm for a continuous period of 150 ms.. As in Experiment 1, we corrected the data for fingers width. To do so, we used the grip aperture between the fingers when subjects grasped the objects (to allow similar tactile feedback as in Experiment 1) following each manual estimation trial. Five participants were removed from the analysis, one due to equipment failure and an additional four due to missing data in one or more of the experimental conditions.

## Results

### Experiment 1: Results and discussion

**Figure 2:**
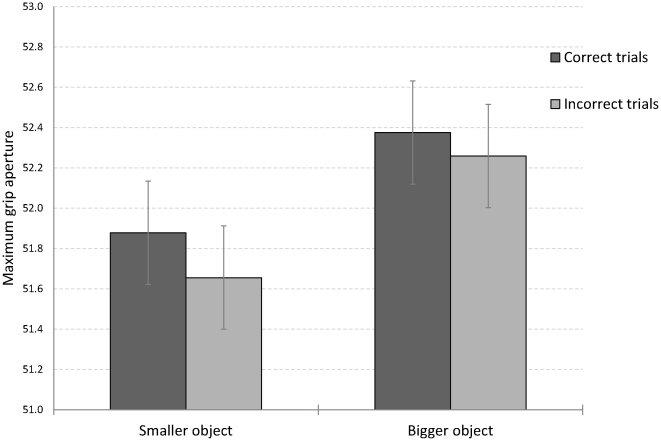
Maximum grip apertures (MGAs) for correct and for incorrect size classifications in Experiment 1. MGAs during grasping reflected the real size differences between the two objects. This was found even in trials in which subjects erroneously judged the larger object in the pair as the smaller one. Error bars denote confidence intervals of the main effect of object size for repeated measures designs [29].

Accuracy of perceptual discrimination was 58.7%, on average. The kinematic data were analyzed separately for trials with correct and with incorrect perceptual discrimination (see Figure 2).

The data depicted in Figure 2 reveal that the MGA faithfully reflected object size - regardless of the accuracy of the perceptual estimation. The MGA was veridical even in cases in which the two objects were not discriminable perceptually. Moreover, the difference in aperture between the fingers reflected the real physical difference in size between the two objects (0.5 mm).

An ANOVA was performed on the MGA data, with experimental block, object size and perceptual judgment accuracy as within-subject variables. Unless otherwise stated, two-tailed tests were used for all statistical analyses. The single reliable main effect was that of object size (F(1,21) = 13.04, p<.01, η_p_^2^ = .38) with larger MGAs for the larger object. Notably, accuracy of perceptual discrimination did not affect the MGAs (F(1,21) = 1.11, p>.1, η_p_^2^ = .05) nor did perceptual discrimination interact with object size (F<1).

For dedicated comparisons, the MGA difference between large and small objects was reliable for both correct and incorrect discrimination (F(1,21) = 6.21, η_p_^2^ = .23 and F(1,21) = 5.19, p<05, η_p_^2^ = .20, respectively). The last result is noteworthy. Even when the participants erroneously judged the physically smaller object to be the larger one, the respective MGAs still faithfully reflected the real difference in size. The results show that accuracy of grasping is independent of accuracy for visual perception.

An additional ANOVA was performed to test for effects of within-trial order of responding revealed neither a main effect of order nor any interactions with order.

**Figure 3:**
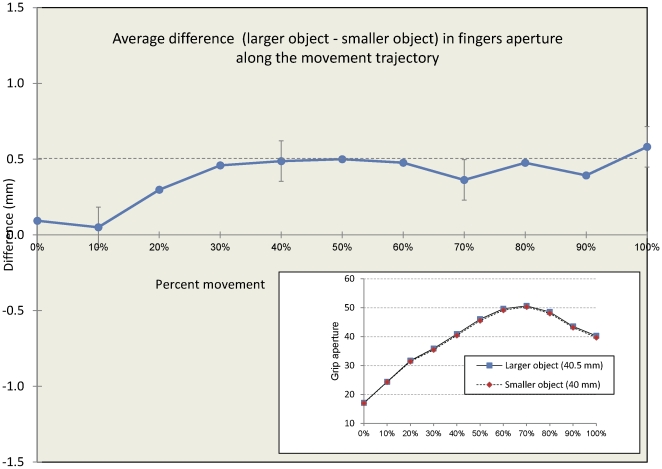
Average difference in aperture between the larger and the smaller object in different segments of the movement. The opening between fingers was tuned to the actual size difference between the objects (dashed line) during most of the movement trajectory. Insert figure shows the average grip aperture data throughout the movement. Error bars denote confidence intervals of the main effect of object size for repeated measures designs [29].

Finally, in order to test whether the grasping fingers were sensitive the small size difference at different stages of the movement [12], we conducted an analysis on changes in grip aperture throughout the movement (for a similar analysis see [17]). The results of this analysis are presented in Figure 3. A directional difference in aperture was apparent between the larger and the smaller object throughout the movement trajectory. This difference reflected the real difference in size of the objects even prior to reaching MGA (which accrued at about 70% of the movement). We performed an additional ANOVA on the movement trajectories, with movement time (0% to 100% in gaps of 10%), object size, and perceptual accuracy as within-subject variables. The main effect of movement time (F(10,210) = 199.8, p<.01, η_p_^2^ = .9) reflected the changes in aperture throughout the movement trajectory, with an increase in aperture peaking at MGA, and followed by a decrease in aperture prior to grasp (see Figure 3, again). The main effect of object size reflected the sensitivity of the fingers to objects of different size throughout the movement (F(1,21) = 7.43, p<.05, η_p_^2^ = .26). The main effect of the perceptual discrimination accuracy was not significant (F<1), as well as the interactions between perceptual discrimination and object size (F(1,21) = 2.54, p>.1, η_p_^2^ = .10), between perceptual discrimination and movement time (F(10,210) = 1.54, p>.1, η_p_^2^ = .07), and between movement time and object size (F(10,210) = 1.13, p>.1, η_p_^2^ = .05). The three-way interaction was not significant as well (F(10,210) = 1.34, p>.1, η_p_^2^ = .06). Planned comparisons showed that the effect of object size was not significant in the first third of the movement (10–30%, F(1,21) = 1.53, p>.1, η_p_^2^ = .07), but was significant in the second (40–60%, F(1,21) = 4.54, p<.05, η_p_^2^ = .18) and in the third portions (70–90%, F(1,21) = 8.21, p<.01, η_p_^2^ = .28) of the movement trajectory.

To test the generality of our results using a different normalization method, a standard trial-by-trial normalization procedure was applied for the movement data, which yielded a similar pattern of results (see Figure S1, Supporting information).

### Experiment 2: Results and discussion

Accuracy of perceptual discrimination was 62.7%, on average. Note that accuracy in Experiment 2 was higher than that in Experiment 1, but was still within the conventional range of uncertainty (25%–75%) considered in psychophysical measures [1], [13], [14]. A possible explanation for this improvement is that the two perceptual tasks were performed in full within each trial in the experiment. To the extent that the two tasks rely on a common perceptual resource, the improvement could be the result of training effects in perceptual discriminations of size.

**Figure 4:**
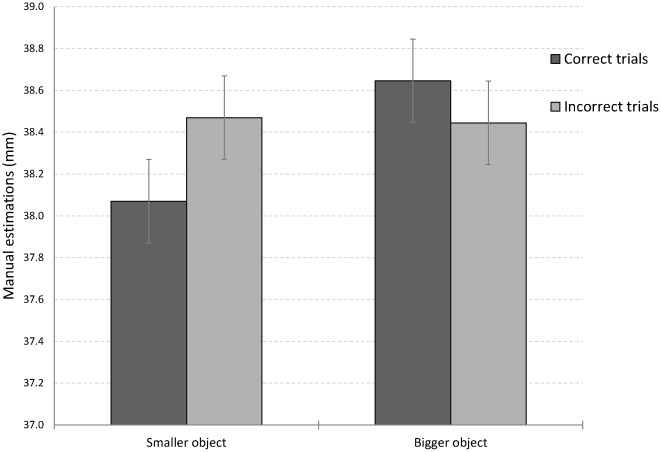
Manual estimations of object's length for correct and incorrect classifications of size in Experiment 2. Manual estimations interacted with size classification judgments. No effect of object size was found for the incorrect trials. Error bars denote confidence intervals of the main effect of object size for repeated measures designs [29].

The kinematic data of the manual estimations were analyzed separately for correct and incorrect trials. As can be seen in Figure 4, the accuracy of the manual estimations was dependent on the accuracy of the perceptual classifications.

The ANOVA on the manual estimations, with block, object size, and perceptual classification accuracy as within-subject variables, showed an effect of block (F(2,48) = 5.3, p<.01, η_p_^2^ = .18) with larger estimations for the first compared to the second and third blocks (39.5, 38.1, and 37.6 mm, respectively). The main effect of object size was marginally significant (F(1,24) = 4.16, p>.05, η_p_^2^ = .15). Note, the unlike the MGAs in Experiment 1, which were obviously larger than the physical size of the objects, manual estimations in Experiment 2 underestimate physical object size, as is typically found for this measure [7].

Most important, object size interacted with the accuracy of the perceptual classification (F(1,24) = 3.66, p<.05, η_p_^2^ = .13, one tailed). Dedicated comparisons showed that the interaction resulted from a significant effect of object size for correct responses (F(1,24) = 11.18, p<.01, η_p_^2^ = .32) in tandem with the absence of an effect of object size for incorrect responses (F<1). Within-trial order of responding did not have an effect except for a four-way interaction with block, object size, and classification accuracy (F(2,46) = 4.05, p>.05, η_p_^2^ = .15).

The findings of Experiment 2 show that the results of Experiment 1 were not confounded by the different response demands of the two tasks. In particular, the continuous perceptual measure of manual estimations was modulated in Experiment 2 by the accuracy of the perceptual discrimination response, whereas a similar continuous measure in the domain of grasping was found to be independent from perceptual discriminations (Experiment 1). These findings show that it was the domain of the response – visually-guided action versus visual perception – that sustained the dissociation found between grasping and perception in Experiment 1.

## Discussion

The current results show that resolution power for grasping is independent of resolution power for perception. The opening between the grasping fingers in Experiment 1 always reflected the actual values of size of the objects regardless of whether the same observers perceived correctly the relative sizes of the objects. These results concur with Goodale and Milner's account of the organization of the primate visual system [18], [19]. According to their idea of two dedicated visual systems, the ventral ‘perception’ pathway provides the rich and detailed representation of the world as we see it, whereas the dorsal ‘action’ pathway enables flexible control of actions directed to objects in the outside environment. This proposal of a functional separation between visual systems underling action and perception has been supported by converging evidence from different domains [9], [20]–[24].

Our findings also concur with the results of behavioral studies showing that visually guided action can be dissociated from conscious perception [3], [8], [9], [25]–[27]. In a different study conducted in our lab, a dissociation was found for a visual illusion [3]. Yet, it is still a matter of debate whether visual illusions provide a solid ground for a dissociation between action and perception [5]–[7], [28]. The present study provides evidence for the dissociation of perception and action in basic psychophysical performance unrelated to the context of visual illusions.

Our findings can be also be interpreted in light of the proposal that vision for action and vision for perception use different visual metrics [5]. According to this view, action is usually based on an observer-relative (absolute) frame of reference, which can be independent of the context in which the object is embedded. Perception, on the other hand, is considered to more heavily rely on a relative frame of reference, in which objects are perceived compared to other objects in their visual context. This view provides an appealing account for the dissociation found between perception and action in Experiment 1, in which the perceptual task emphasized direct comparisons between the two objects. The interpretation of the results of Experiment 2 in terms of different frames of reference is less trivial, however, because the manual estimation task did not emphasize relative processing but rather independent estimations of the target object. Still, manual estimations in Experiment 2 were affected by the perceptual decision, which argues that our effects reflect the nature of the task in hand (e.g., perception vs. action) rather than specific task requirements.

The agreed estimation of the Weber fraction for visual length has been 3% [15]. In this study, we used two stimuli with a size difference of about 1%. Despite the small difference in size, the opening between the fingers still accurately reflected the actual size of the objects. Our data show that human resolution power is not captured fully by the classic perceptual methods. Grasping can exhibit greater resolution power than that based on conscious visual perception alone.

## Supporting Information

Figure S1 To test the generality of our results, a standard trial-by-trial normalization procedure was applied for the movement data of Experiment 1.Each data point in each trial was normalized by the initial opening between the fingers in this trial (from which the size of the start button was subtracted). As can be seen in the figure, the new analysis yielded a similar pattern of results to the one obtained in Figure 3. Yet, due to the inclusion of an additional source of noise driven by trial-by-trail variability, weaker statistical effects were obtained. Simple comparisons showed that the effect of object size was not significant in the first third of the movement (10–30%, F(1,21)<1), was marginally significant in the second portion (40–60%, F(1,21) = 2.83, p = .053, ηp2 = .12, one tailed) and was significant in the third portion (70–90%, F(1,21) = 5.77, p<.05, ηp2 = .22) of the movement trajectory.
(TIF)
