# Supplementary figures and images for: Accurate Visuomotor Control below the Perceptual Threshold of Size Discrimination
Source: PLoS One. 2012 Apr 27;7(4):e36253. doi: 10.1371/journal.pone.0036253 (PMC3338698; doi:10.1371/journal.pone.0036253)

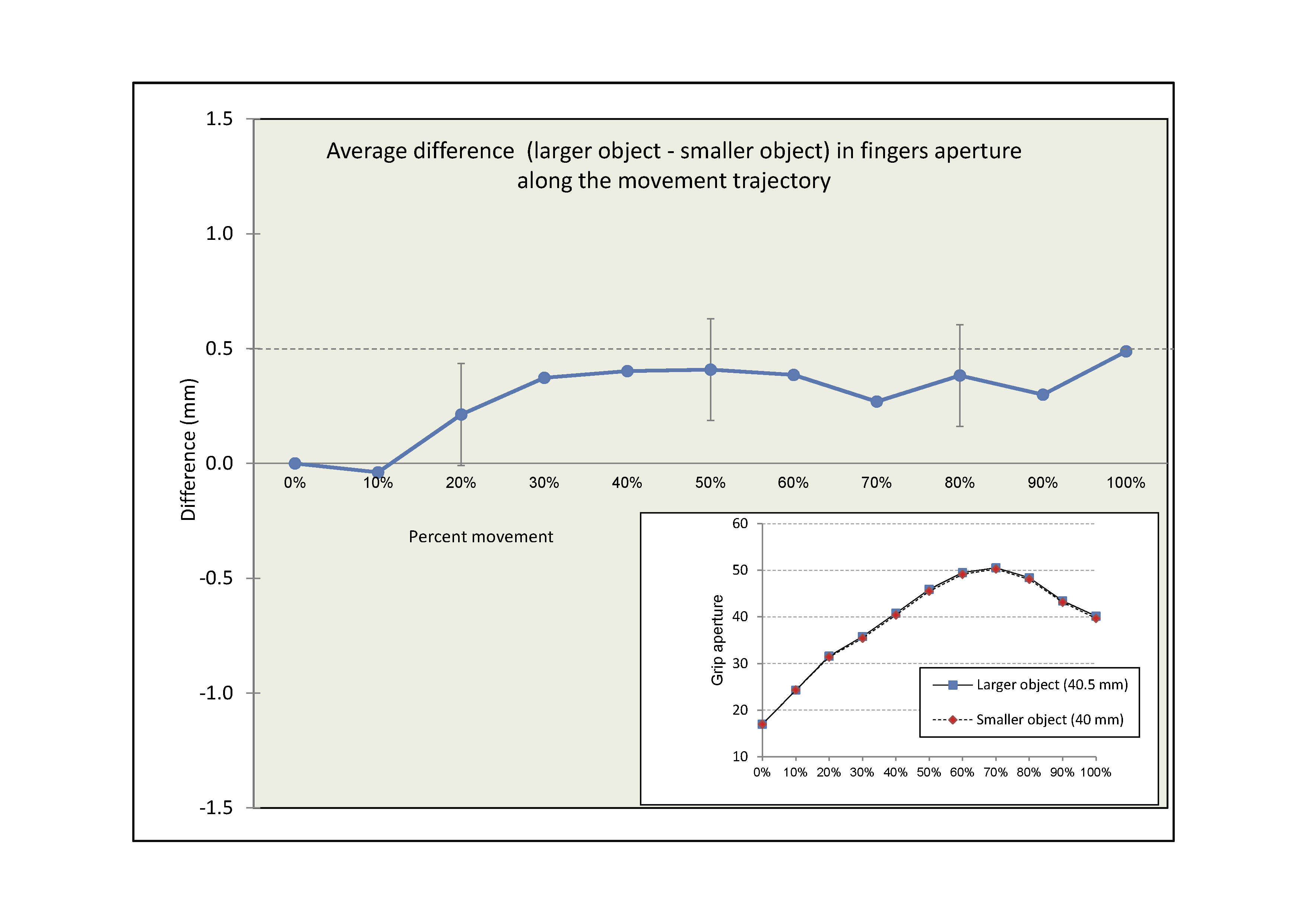

Supplement: Figure S1 — To test the generality of our results, a standard trial-by-trial normalization procedure was applied for the movement data of Experiment 1.Each data point in each trial was normalized by the initial opening between the fingers in this trial (from which the size of the start button was subtracted). As can be seen in the figure, the new analysis yielded a similar pattern of results to the one obtained in Figure 3. Yet, due to the inclusion of an additional source of noise driven by trial-by-trail variability, weaker statistical effects were obtained. Simple comparisons showed that the effect of object size was not significant in the first third of the movement (10–30%, F(1,21)<1), was marginally significant in the second portion (40–60%, F(1,21) = 2.83, p = .053, ηp2 = .12, one tailed) and was significant in the third portion (70–90%, F(1,21) = 5.77, p<.05, ηp2 = .22) of the movement trajectory. (TIF) [file pone.0036253.s001.tif]
